# Reproducibility of infant fNIRS studies: a meta-analytic approach

**DOI:** 10.1117/1.NPh.10.2.023518

**Published:** 2023-03-08

**Authors:** Jessica Gemignani, Irene de la Cruz-Pavía, Anna Martinez, Caroline Nallet, Alessia Pasquini, Gaia Lucarini, Francesca Cavicchiolo, Judit Gervain

**Affiliations:** a University of Padua, Department of Developmental and Social Psychology, Padua, Italy; b University of Padua, Padova Neuroscience Center, Padua, Italy; c University of the Basque Country, Department of Linguistics and Basque Studies, Vitoria-Gasteiz, Spain; d Ikerbasque, Basque Foundation for Science, Bilbao, Spain; e Université Paris Cité & CNRS, Integrative Neuroscience and Cognition Center, Paris, France

**Keywords:** replication crisis, infant NIRS data, meta-analysis, rule learning

## Abstract

**Significance:**

Concerns about the reproducibility of experimental findings have recently emerged in many disciplines, from psychology to medicine and neuroscience. As NIRS is a relatively recent brain imaging technique, the question of reproducibility has not yet been systematically addressed.

**Aim:**

The current study seeks to test the replicability of effects observed in NIRS experiments assessing young infants’ rule-learning ability.

**Approach:**

We conducted meta-analyses and mixed-effects modeling-based inferential statistics to determine whether effect sizes were replicable and comparable in a sample of 23 NIRS studies investigating infants’ abilities to process repetition- and diversity-based regularities in linguistic and nonlinguistic auditory and visual sequences. Additionally, we tested whether effect sizes were modulated by different factors such as the age of participants or the laboratory. We obtained NIRS data from 12 published and 11 unpublished studies. The 23 studies involved a total of 487 infants, aged between 0 and 9 months, tested in four different countries (Canada, France, Italy, and USA).

**Results:**

Our most important finding is that study and laboratory were never significant moderators of variation in effect sizes, indicating that results replicated reliably across the different studies and labs included in the sample. We observed small-to-moderate effect sizes, similar to effect sizes found with other neuroimaging and behavioral techniques in the developmental literature. In line with existing findings, effect sizes were modulated by the participants’ age and differed across the different regularities tested, with repetition-based regularities giving rise to the strongest effects; in particular, the overall magnitude of this effect in the left temporal region was 0.27 when analyzing the entire dataset.

**Conclusions:**

Meta-analysis is a useful tool for assessing replicability and cross-study variability. Here, we have shown that infant NIRS studies in the language domain replicate robustly across various NIRS machines, testing sites, and developmental populations.

## 1 Introduction

Since the introduction of near-infrared spectroscopy, its use has grown dramatically in the neurosciences, particularly in developmental cognitive neuroscience,^1^^,^^2^ due to its many practical advantages: ease of use, portability, noninvasiveness, and low cost.

As the volume of fNIRS research in developmental neuroscience has increased, so have the concerns about the reproducibility of experimental findings, a core principle of scientific progress. Issues about reproducibility have emerged in many related areas, from psychology to medicine, as some key findings were found not to replicate reliably.^3^^–^^6^ Reasons for the replication crisis include undocumented diversification in research methodology and data analysis techniques, nontransparent data inclusion and exclusion criteria, difficulty publishing null results, HARKing (hypothesizing after the results are known), p-hacking, and other dubious analysis and reporting practices. Some of these issues are further exacerbated in developmental research, as young children are challenging participants, which may lead to large amounts of missing data, unclear data rejection criteria, short study durations and thus small numbers of trials per participant, small sample sizes, and relatedly, low statistical power. Developmental NIRS research is no exception, especially because NIRS is a relatively new technique, and standardization of research practices has only started recently,^7^ e.g., with systematic comparisons of handware performance,^8^^,^^9^ preprocessing methods,^10^^–^^12^ and statistical analyses.^13^^,^^14^

NIRS by now has become a very commonly used neuroimaging technique in developmental neuroscience. It is thus timely to address the question of reproducibility in a systematic way to support good theory-building and to identify issues that weaken replicability.

One approach to assessing replicability, quantifying cross-lab variability and identifying potential moderating factors underlying variability is to conduct a meta-analysis of existing (published and/or unpublished) studies that address the same research question. A meta-analysis is a quantitative method for aggregating across experimental studies^15^ to reveal the average effect size of a specific phenomenon. In addition to quantifying replicability and variability across studies, meta-analyses have the advantage of pooling data over a larger sample size than typically possible in single studies, licensing more robust or more general conclusions.^16^ Meta-analyses have been conducted over various types of data in many different domains of psychology, neuroscience, and medicine^17^^–^^22^ and are readily applicable to developmental data.^23^

The goal of the current study is, therefore, to conduct a meta-analysis of infant NIRS studies in an attempt to test for reproducibility, quantify variability across studies, and identify moderators explaining the observed variability. We chose repetition-based rule learning as the phenomenon under investigation. The ability to extract and learn rules from speech is foundational for language development.^24^ This question has thus received considerable attention in the language acquisition literature. In particular, a large number of behavioral and NIRS studies (for reviews, Refs. 25 and 26, respectively) asked whether infants are able to recognize and represent different sequences that include a repetition (e.g., AAB versus ABB) or distinguish them from diversity-based sequences, i.e., sequences in which all syllable are different (e.g., ABB versus ABC). These studies typically use artificial grammars that generate sequences with the relevant structure (e.g., ABB: “mubaba,” “penana,” etc., ABC: “mubage,” “penaku,” etc.).

We chose this research question because a relatively large number of published and unpublished NIRS studies addressed it using similar stimuli and experimental designs. Such a sample of studies allows for a good estimation of lab-based variation, e.g., differences in machine type, experimenter characteristics, NIRS-relevant population characteristics, such as hair density and color, etc. Many of these factors are not under the researchers’ direct control and often remain unreported in publications. This is the type of variation that meta-analyses are particularly well suited to assess.

Ours is the first meta-analytic study of infant NIRS data, and its aim is primarily methodological. We seek to provide a first quantitative assessment of variability in effect sizes across NIRS studies that differ along specific dimensions to address the issue of cross-lab/cross-study variability. In other words, we are less concerned here with the theoretical issues related to infants’ ability to learn linguistic rules, despite its relevance for developmental research. For these questions, we refer the reader to Refs. 25 and 26.

## 2 Methods

### 2.1 Data

#### 2.1.1 Studies

We aggregated 23 published and unpublished fNIRS studies, conducted in four different laboratories, testing brain responses of typically developing infants to two different types of linguistic regularities: repetition-based regularities (R) and diversity-, i.e., nonrepetition-based regularities (N). The studies were identified by searching through PubMed and Google Scholar, using the search strings “repetition-based regularity,” “rule learning,” “fNIRS,” and “infants.” Exclusion criteria included (i) testing atypical populations or (ii) using methods other than NIRS. Papers including more than one study were considered separate studies. Of the 43 hits, those that met either of the exclusion criteria were discarded, leaving 12 published studies.^27^^–^^31^ Additionally, 11 studies from the last author’s laboratory were added. These studies were not published in peer-reviewed articles, although some of them are available online in PhD dissertations (Table 1). We know of no other unpublished studies.

**Table 1:** List of studies included in each of the three meta-analyses (R versus 0, N versus 0, and R versus N). As described in Sec. 2.1.1, different sets of studies contributed to the three comparisons of interest, indicated by the color of the last three columns (bold = included, NA = not included); the columns also report which regularity was considered for each study.

ID	Study	Publication	Cond 1	Cond 2	Adjacency of the repetition	Position of the repetition	Modality of input	Type of input	Age (month)	Sample size	Lab	Included in which statistical analysis		
												R*vs*0	N*vs*0	R*vs*N
1	0m-Speech-A_A-A_C	Gervain PhD thesis	A_A	A_C	Adjacent	NA	Auditory	Linguistic	0	21	Trieste	**A_A**	**A_C**	**A** **_** **A**
2	0m-Speech-AA-AB	Gervain PhD thesis	AA	AB	Adjacent	NA	Auditory	Linguistic	0	22	Trieste	**AA**	**AB**	**AA**
3	6m-VisualNonLinguistic- AA-AB	Berent et al. (2021)	AA	AB	Adjacent	NA	Visual	NonLinguistic	6	21	Paris	**AA**	**AB**	**AA**
4	6m-VisualLinguistic- AA-AB	Berent et al. (2021)	AA	AB	Adjacent	NA	Visual	Linguistic	6	23	Paris	**AA**	**AB**	**AA**
5	6m-Speech-AA-AB	Unpublished	AA	AB	Adjacent	NA	Auditory	Linguistic	6	15	Paris	**AA**	**AB**	**AA**
6	0m-Speech-AAB-ABC	Gervain et al. (2012)	AAB	ABC	Adjacent	Initial	Auditory	Linguistic	0	22	Vancouver	**AAB**	**ABC**	**AAB**
7	0m-Speech-AAB-ABB- AltNonAlt- R=AAB	Gervain et al. (2012)	AAB	ABB	Adjacent	Initial	Auditory	Linguistic	0	20	Vancouver	**AAB**	NA	NA
8	0m-Speech-AAB-ABB- AltNonAlt- R=ABB	Gervain et al. (2012)	AAB	ABB	Adjacent	Final	Auditory	Linguistic	0	20	Vancouver	**ABB**	NA	NA
9	0m-Speech-AAB-ABB- SimpleBlocks- R=AAB	Gervain et al. (2012)	AAB	ABB	Adjacent	Initial	Auditory	Linguistic	0	24	Vancouver	**AAB**	NA	NA
10	0m-Speech-AAB-ABB- SimpleBlocks- R=ABB	Gervain et al. (2012)	AAB	ABB	Adjacent	Final	Auditory	Linguistic	0	24	Vancouver	**ABB**	NA	NA
11	0m-Speech-AAB-ABC- ReplicationNIRx	Unpublished	AAB	ABC	Adjacent	Initial	Auditory	Linguistic	0	24	Paris	**AAB**	**ABC**	**AAB**
12	0m-Tones-AAB-ABC	Unpublished	AAB	ABC	Adjacent	Initial	Auditory	NonLinguistic	0	20	Paris	**AAB**	**ABC**	**AAB**
13	6m-Speech-AAB-ABC- Entropy	Radulescu et al. (in prep)	AAB	ABC	Adjacent	Initial	Auditory	Linguistic	6	21	Paris	**AAB**	**ABC**	**AAB**
14	0m-Speech-ABA-ABC	Gervain et al. 2008	ABA	ABC	NonAdjacent	NA	Auditory	Linguistic	0	22	Trieste	**ABA**	**ABC**	**ABA**
15	0m-Speech-ABB-ABC	Gervain et al. 2008	ABB	ABC	Adjacent	Final	Auditory	Linguistic	0	22	Trieste	**ABB**	**ABC**	**ABB**
16	0m-Speech-ABB-ABC- RepeatedContext-R=C	Bouchon PhD thesis	ABB	ABC	Adjacent	Final	Auditory	Linguistic	0	24	Paris	**ABB**	**ABC**	**ABB**
17	0m-Speech-ABB-ABC- RepeatedContext-R=V	Bouchon PhD thesis	ABB	ABC	Adjacent	Final	Auditory	Linguistic	0	24	Paris	**ABB**	NA	**ABB**
18	0m-Speech-ABB-ABC- VariableContext-R=C	Bouchon PhD thesis	ABB	ABC	Adjacent	Final	Auditory	Linguistic	0	21	Paris	**ABB**	**ABC**	**ABB**
19	0m-Speech-ABB-ABC- VariableContext-R=V	Bouchon PhD thesis	ABB	ABC	Adjacent	Final	Auditory	Linguistic	0	21	Paris	**ABB**	**NA**	**ABB**
20	0m-Speech-ABB-ABC-CV	Bouchon et al. (2015)	ABB	ABC	Adjacent	Final	Auditory	Linguistic	0	24	Paris	**ABB**	**ABC**	**ABB**
21	6m-Speech-ABB-ABC- AltNonAlt	de la Cruz-Pavía and Gervain (under review)	ABB	ABC	Adjacent	Final	Auditory	Linguistic	6	24	Paris	**ABB**	**ABC**	**ABB**
22	7m-Speech-ABB-ABC	Wagner et al. (2011)	ABB	ABC	Adjacent	Final	Auditory	Linguistic	7	13	Boston	**ABB**	**ABC**	**ABB**
23	9m-Speech-ABB-ABC	Wagner et al. (2011)	ABB	ABC	Adjacent	Final	Auditory	Linguistic	9	15	Boston	**ABB**	**ABC**	**ABB**

The studies used similar methodology, e.g., similar stimuli and experimental designs, but it addressed slightly different theoretical questions and, as a result, varied along a few dimensions (e.g., the auditory versus visual nature of the stimuli, see Table 1). We used these factors as moderators in the meta-analysis. Furthermore, studies varied in whether they tested repetition-based regularities, diversity-based regularities, or both (Table 1). Consequently, we conducted three separate meta-analyses evaluating the effect sizes of (i) the comparison between brain responses to repetitions versus a zero baseline (“R versus 0”); (ii) the comparison between brain responses to diversity (nonrepetition) versus a zero baseline (“N versus 0”); and (iii) the comparison between brain responses to repetitions versus diversity (“R versus N”). A specific study may have contributed to just one or several of the comparisons (see the last column of Table 1). As a result, 23 studies were included in the final analysis for the R versus 0 comparison, 19 in the analysis of the R versus N comparison, and 17 in the analysis of the N versus 0 comparison. Details can be found in Table 1. Additionally, Table 2 reports the most relevant technical details of NIRS data acquisition.

**Table 2:** Technical characteristics of the NIRS machines employed in each study.

ID	Study	Lab	CW-NIRS	Fs (Hz)	Wavelengths (nm)
1	0m-Speech-A_A-A_C	Trieste	Hitachi ETG-4000	10	695, 830
2	0m-Speech-AA-AB	Trieste	Hitachi ETG-4000	10	695, 830
3	6m-VisualNonLinguistic-AA-AB	Paris	NIRx NIRScout 8x16	15.625	760, 850
4	6m-VisualLinguistic-AA-AB	Paris	NIRx NIRScout 8x16	15.625	760, 850
5	6m-Speech-AA-AB	Paris	NIRx NIRScout 8x16	15.625	760, 850
6	0m-Speech-AAB-ABC	Vancouver	Hitachi ETG-4000	10	695, 830
7	0m-Speech-AAB-ABB-AltNonAlt-R=AAB	Vancouver	Hitachi ETG-4000	10	695, 830
8	0m-Speech-AAB-ABB-AltNonAlt-R=ABB	Vancouver	Hitachi ETG-4000	10	695, 830
9	0m-Speech-AAB-ABB-SimpleBlocks-R=AAB	Vancouver	Hitachi ETG-4000	10	695, 830
10	0m-Speech-AAB-ABB-SimpleBlocks-R=ABB	Vancouver	Hitachi ETG-4000	10	695, 830
11	0m-Speech-AAB-ABC-ReplicationNIRx	Paris	NIRx NIRScout 8x16	10	760, 850
12	0m-Tones-AAB-ABC	Paris	Hitachi ETG-4000	10	695, 830
13	6m-Speech-AAB-ABC-Entropy	Paris	NIRx NIRScout 8x16	15.625	760, 850
14	0m-Speech-ABA-ABC	Trieste	Hitachi ETG 4000	10	695, 830
15	0m-Speech-ABB-ABC	Trieste	Hitachi ETG 4000	10	695, 830
16	0m-Speech-ABB-ABC-RepeatedContext-R=C	Paris	NIRx NIRScout 10x8	10.417	760, 850
17	0m-Speech-ABB-ABC-RepeatedContext-R=V	Paris	NIRx NIRScout 10x8	10.417	760, 850
18	0m-Speech-ABB-ABC-VariableContext-R=C	Paris	NIRx NIRScout 10x8	10.417	760, 850
19	0m-Speech-ABB-ABC-VariableContext-R=V	Paris	NIRx NIRScout 10x8	10.417	760, 850
20	0m-Speech-ABB-ABC-CV	Paris	NIRx NIRScout 10x8	10.417	760, 850
21	6m-Speech-ABB-ABC-AltNonAlt	Paris	NIRx NIRScout 8x16	15.625	760, 850
22	7m-Speech-ABB-ABC	Boston	Hitachi ETG-4000	10	695, 830
23	9m-Speech-ABB-ABC	Boston	Hitachi ETG 4000	10	695, 830

The study comprised data from a total of 487 infants, aged between 0 and 9 months, tested in four different countries (Canada, France, Italy, and USA). Table 1 provides information about each study’s individual sample size.

#### 2.1.2 Materials

All included studies used two artificial grammars in the form of bisyllablic or trisyllabic sequences. Table 1 reports the specific structures employed in each study. Other stimulus characteristics can be found in the respective publications and were highly similar across studies.

#### 2.1.3 Procedure

In all studies, infants were tested with an NIRS device (the brand, wavelengths, and sampling frequencies are listed in Table 2), and sound stimuli were administered through two loud speakers.

**Fig. 1:**
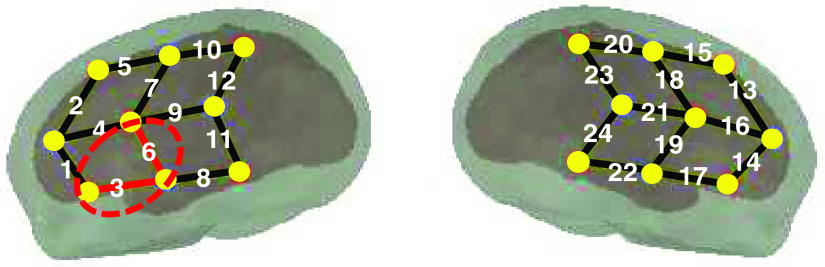
Optode arrangement employed in the studies included 8 or 10 sources and 8 detectors, forming a total of 20 or 24 channels. The region of interest that we focused on is the left temporal lobe, formed by channels 3 and 6; it is highlighted in red.

Eight to ten sources and eight detectors were placed on the infants’ heads bilaterally, with a 2.5 to 3 cm source–detector distance, forming 10 to 12 channels per hemisphere (Fig. 1). The anatomical localization of the resulting array is described in detail in Ref. 32. On the basis of the original study^28^ that first tested newborns’ abilities to detect repetition-based sequences, and given the well-documented relevance of this area for speech and language processing, we focused on the left temporal lobe, which we operationalized as the cluster of channels 3 and 6 (Fig. 1). Choosing the most relevant region of interest (ROI) is a research question in and of itself, with both anatomical and functional localization methods now available in the literature.^7^ Although this issue is relevant for studies comparing different datasets and headgears, it falls outside the scope of the current study. Here, we have chosen to use a predefined ROI, the left temporal region. This area is activated in the large majority of the included studies. Further research will need to address other, study-specific methods for determining the ROI for a meta-analysis.

### 2.2 Data Analysis

#### 2.2.1 fNIRS preprocessing

For studies 1 to 21 (Table 1), NIRS data was preprocessed in the same way as in the original publications (or sources, e.g., unpublished PhD dissertations), which was similar across most studies. Briefly, light intensities were converted to optical densities and to hemoglobin concentration changes using the modified Beer–Lambert Law with the absorption coefficients $\mu_{a}$, ${\mathrm{mm}}^{-1}\times {\mathrm{mM}}^{-1}$: $\mu_{a}(\mathrm{HbO},695\;\mathrm{nm})=0.0955$, $\mu_{a}(\mathrm{HbO},760\;\mathrm{nm})=0.1496$, $\mu_{a}(\mathrm{HbO},830\;\mathrm{nm})=0.2320$, $\mu_{a}(\mathrm{HbO},850\;\mathrm{nm})=0.2526$; $\mu_{a}(\mathrm{HbR},695\;\mathrm{nm})=0.4513$, $\mu_{a}(\mathrm{HbR},760\;\mathrm{nm})=0.3865$, $\mu_{a}(\mathrm{HbR},830\;\mathrm{nm})=0.1792$; and $\mu_{a}(\mathrm{HbR},850\;\mathrm{nm})=0.1798$. The product of the optical pathlength and the differential pathlength factor was set to 1, resulting in concentration changes being expressed in mM×mm. A bandpass filter between 0.01 and 0.7 Hz was applied to concentration changes using an fft digital filter. Then, as illustrated in Ref. 10, blocks of single-trial data were rejected if they contained motion artifacts or if the light intensity reached the saturation value, with motion artifacts defined as signal changes larger than 0.1  mM×mm over 0.2 s. The artifact detection and trial rejection procedures were performed independently for each channel, and channels with less than at least two valid blocks were discarded from the analysis. The trial inclusion rate for each study ranged between 52% and 100% (M: 65.1%, SD: 12.8%). Finally, for the nonrejected blocks, a baseline was linearly fit between the mean of the 5 s preceding the onset of the block and the mean of the 5 s preceding the onset of the next one. Blocks were then averaged within each infant to obtain channel-wise block averages for each condition as well as across infants to obtain grand averages. This preprocessing routine has been shown to yield an accurate recovery of the infant hemodynamic response.^10^ Study-level grand averages were employed to compute study-level effect sizes, whereas individual trial averages were employed to compute infant-level effect sizes (Sec. 2.2.2).

For studies 22 and 23 (Table 1), we obtained each subject’s channel-wise average activation to each experimental condition, i.e., we obtained preprocessed data from the authors and had no access to the raw data. For these studies, we could, therefore, only compute the study-level effect size but not the individual infant-level effect size. Data processing for these datasets is described in the original publication.^27^

#### 2.2.2 Calculation of effect sizes

We performed two analyses using two different and complementary approaches: a meta-analytical approach that analyzes study-level effect sizes and a mixed-effects modeling approach that analyzes infant-level effect sizes. The meta-analytic framework estimates the variability of effect sizes across studies. It can be conducted even when only group-level averages, but not individual participant data, are available and when procedures or data types are not standardized.^23^ When trial-level data for each participant is available, it is possible to compute individual effect sizes and perform a mixed-effects model, yielding a more sensitive measure of within-study variability. In the current study, participant-level NIRS data was available for 21 of the 23 included studies, and it accounted for the entirety of two age groups (newborns and 6-month-olds).

We conducted both meta-analyses and mixed-effects models for the 21 studies for which individual trial-level data was available, whereas the two studies for which we only had individual averages were only entered into the meta-analysis. We did not use NIRS data as the dependent variable in either the meta-analyses or the mixed-effects models because relative concentrations, which are the NIRS measures obtained in the included studies, are not necessarily comparable across different participants and different machines. We used effect sizes as the dependent variable instead.

For both analyses, we first averaged the time series of the hemodynamic response within a block over a time window starting at the onset of the stimulus and lasting up to 15 s after the end of the stimulation block. Effect sizes were then computed for three comparisons of interest: (i) R versus 0, i.e., the comparison between the repetition condition and the zero baseline; (ii) N versus 0, i.e., the comparison between the nonrepetition condition and the zero baseline; (iii) R versus N, i.e., a comparison between repetition and nonrepetition responses.

**Fig. 2:**
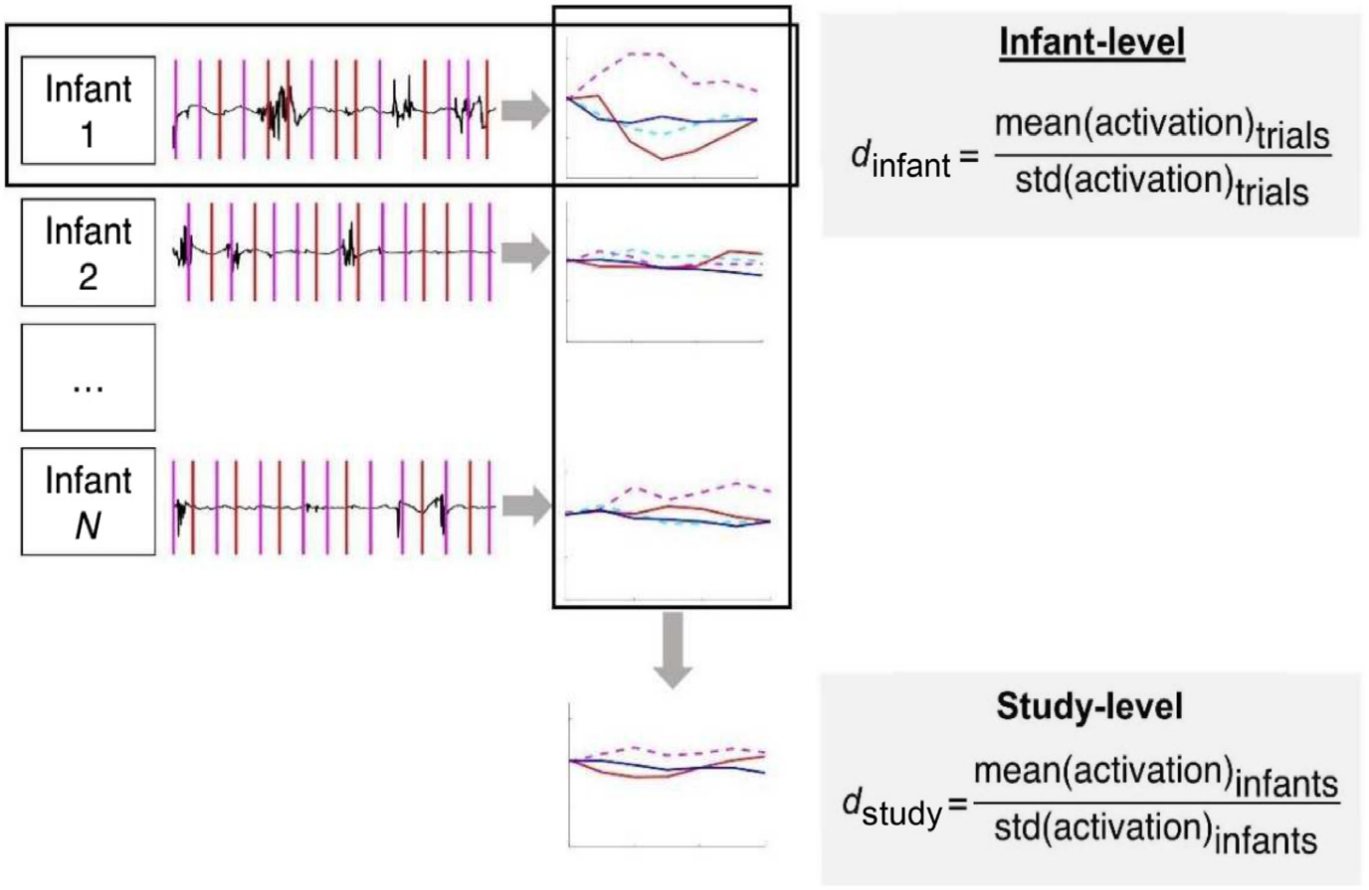
Schematic illustration of infant-level and study-level effect sizes calculations. Activation refers to the R versus 0, N versus 0, and R versus N contrasts computed as the average of the HRF along its time course. Magenta and cyan indicate repetition trials (HbO and HbR, respectively), and red and blue indicate nonrepetition trials (HbO and HbR, respectively).

The meta-analytic effect sizes ($d_{\text{study}}$) were computed by averaging a participant’s responses in all trials of a given condition (Fig. 2). These individual means were then averaged and divided by their standard deviation. The sampling variances of the meta-analytic effect sizes were computed as $V_{d}=2/n+d_{\text{study}}^{2}/4n$, with n being the number of participants^15^; i.e., effect sizes were weighted by the number of participants in a study.^33^

The individual effect sizes ($d_{\text{infant}}$) were computed by dividing each infant’s average activation in a given condition by its standard deviation across trials (Fig. 2).

Both individual and meta-analytic effect sizes were calculated for each channel and hemoglobin component independently. Then, we averaged effect sizes across channels within our predefined ROI, i.e., the left temporal area.

#### 2.2.3 Statistical analysis

##### Meta-analysis

Study-level effect sizes were analyzed by employing meta-analytic random-effects models with the *metafor* package in R.^34^ Models were fit using restricted maximum likelihood (REML).

In addition to this overall meta-analysis, we also conducted moderated meta-analyses to test the effects of specific factors on cross-study effect sizes and variability. Specifically, we explored whether the effect was significantly moderated by the lab (Boston/Trieste/Paris/Vancouver), as this allowed us to directly address our main question of cross-lab replicability. We also tested the moderator age with four levels (newborns/6-/7-/9-months). Infants’ ability to learn repetition-based regularities has been reported to improve between 5 and 11 months^35^^,^^36^ at the behavioral level, and by 6 months of age, infants show much stronger brain responses to diversity-based regularities than at birth.^26^ The ability to process these structural regularities may thus undergo developmental change, which we sought to model by adding age as a moderator to the meta-analyses.

Finally, we analyzed whether the effect size was moderated by repetition position within trisyllabic sequences (initial/final). Existing results suggest that final repetitions may be easier to process than initial ones, possibly due to recency effects in memory, although this advantage may be relatively weak.^37^ We thus included repetition position for the comparisons in which it was relevant, namely R versus 0 and R versus N.

Importantly, these three analyses explored each moderating variable independently, and their joint impact on the effect was analyzed through mixed-effects modeling (see below).

Although our dataset included both published and unpublished studies, for the sake of completeness, we also generated funnel plots and the fail-safe-N as estimates of publication bias.^34^ Funnel plots can identify certain forms of publication bias. Estimates are expected to be randomly sampled around the mean if there is no bias in the published or selected studies, whereas an asymmetry in the distribution of the effects may indicate either true heterogeneity in the phenomenon under study or that studies with nonsignificant findings remained unpublished.^16^ The fail-safe-N is another useful measure to detect bias in the literature as it quantifies how many unpublished studies with null results would have to exist for the overall effect size to be zero. We computed the funnel plots and the fail-safe-N using the *metafor* R package,^34^ following recommendations in the literature.^38^

##### Linear mixed-effects modeling

Linear mixed-effects models were computed over infant-level effect sizes. The planned random effects structure consisted of random intercepts for study, lab, and age. In the case of failure to converge, it was iteratively simplified by pruning first the random intercept for age, then for lab, and finally for study. Candidate fixed effects were incrementally included in the fixed effects structure, and the resulting models were compared. The best-fitting model was chosen based on the AIC (Akaike Information Criterion) value.

##### Application of analyses to study sets

**Fig. 3:**
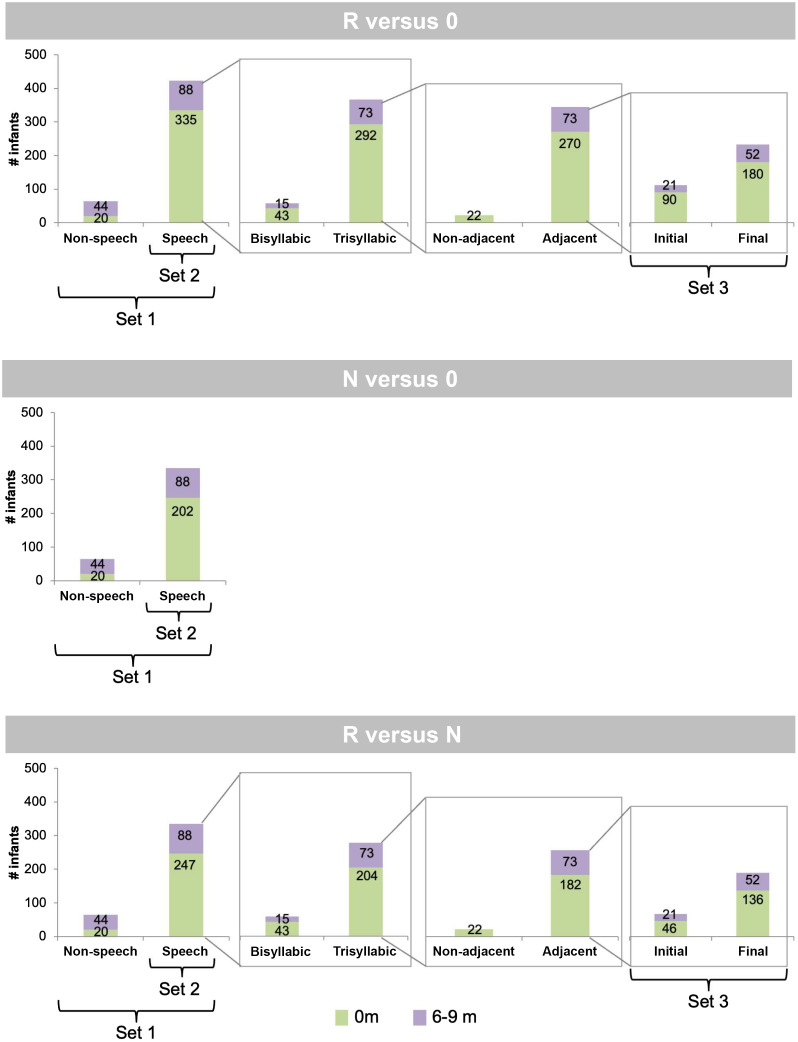
Organization of the dataset into the three sets of studies. The numbers overlaid on the bars indicate the number of participants in each category. Age is color coded (green is newborns, and violet is 6- to 9-month-olds).

Analyses were performed over different sets of studies as a function of the factors to which a given study contributed (Table 1). Specifically, three sets were created on the basis of sample sizes (Fig. 3): (i) the entire dataset (Set 1; R versus 0: 23 studies, N versus 0: 17 studies, R versus N: 19 studies); (ii) a set of studies using speech stimuli (Set 2; R versus 0: 20 studies, N versus 0: 14 studies, R versus N: 16 studies); and (iii) a set of studies using speech stimuli with adjacent repetitions (Set 3; R versus 0: 16 studies, R versus N: 13 studies). The meta-analytic approach was applied to all studies, and mixed-effects modeling was employed only for studies for which raw data was available (studies 1 to 21).

## 3 Results

### 3.1 Meta-Analysis

#### 3.1.1 Unmoderated models

##### R versus 0

**Fig. 4:**
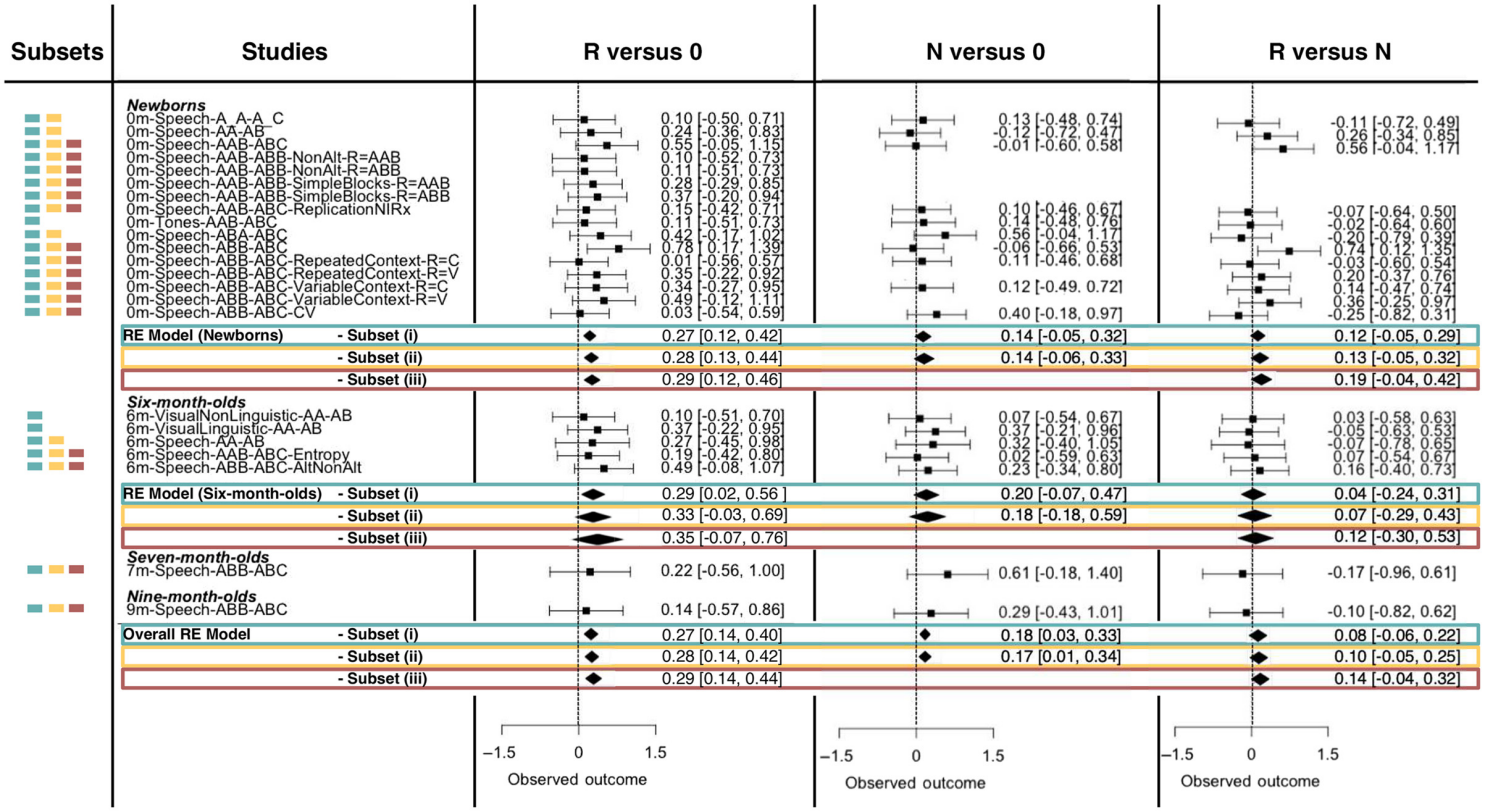
Forest plots of the meta-analytic effect sizes for brain activation in the left temporal area to repetition-based (R versus 0) and diversity-based (N versus 0) regularities, as well as for the difference in activation between them (R versus N) for HbO. Each study’s estimate is indicated by the corresponding square. Error bars indicate the 95% confidence interval. Diamonds show the summary estimates of each set of studies, with the center of the diamond corresponding to the estimate and the left and right edges indicating the confidence interval limits. Not all studies contributed to all comparisons, as described in Sec. 2.1.1 and Table 1. Corresponding forest plots obtained on HbR time traces are reported in the Supplementary Material (Fig. S1).

When analyzing the entire dataset (set 1), the overall magnitude of the effect was 0.271 (95% CI=[0.144,0.398], z=4.20, p<0.001). Breaking it down by age, the effect size was 0.273 for newborns, 0.289 for 6-month-olds, and 0.266 for 7- to 9-month-olds. When considering studies employing speech stimuli (Set 2), the meta-analytic effect size was 0.282 ([0.146, 0.418], z=4.07, p<0.001, 0.282 for newborns, 0.327 for 6-month-olds, and 0.28 for 7- to 9-month-olds). For studies with adjacent trisyllabic repetitions in speech (set 3), the overall magnitude of the effect was 0.288 ([0.136, 0.439], z=3.73, p<0.001), with an effect size of 0.289 for newborns, 0.348 for 6-month-olds, and 0.283 for 7- to 9-month-olds. Corresponding forest plots are shown in the left panel of Fig. 4.

##### N versus 0

The overall magnitude of the effect, computed on the entire dataset, was 0.18 (95% CI = [0.03, 0.33], z=2.35, p<0.05). Estimates were 0.14 for newborns, 0.20 for 6-month-olds, and 0.25 for 7- to 9-month-olds. For Set 2, the estimated meta-analytic effect size was 0.17 (95% CI = [0.01, 0.34], z=2.074, p<0.05), with 0.14 for newborns, 0.18 for 6-month-olds, and 0.26 for 7- to 9-month-olds (Fig. 4).

##### R versus N

The overall magnitude of the effect was 0.08 (95% CI=[−0.06,0.22], z=1.14, ns), with a larger effect for newborns than for older infants (newborns: 0.12, 6-month-olds: 0.04, 7- to 9-month-olds: 0). For set 2, we found an overall meta-analytic effect size of 0.10 (95% CI=[−0.05,0.25], z=1.28, ns), with estimates of 0.133 for newborns, 0.072 for 6-month-olds, and 0.01 for 7- to 9-month-olds (Fig. 4). For set 3, the overall effect size was 0.14 (95% CI=[−0.04,0.32], z=1.56, *ns*), with subgroup estimates that were again larger in newborns (0.19) than for older infants (6-month-olds: 0.12, 7- to 9-month-olds: 0.02).

#### 3.1.2 Moderated analyses

##### R versus 0

The lab-moderated models yielded a nonsignificant effect of lab in the three sets of data, as did the age-moderated models for age. Crucially, lab and age were highly collinear because some ages were only tested in some labs. Therefore, these two moderating effects are better interpreted when analyzed jointly through mixed-effects modeling (Sec. 3.2). In set 3, the only set in which this moderator was analyzed, the model yielded no significant effect of repetition position.

##### N versus 0

The lab-moderated models yielded a nonsignificant effect of lab in all sets of data, as did the age-moderated models for age.

##### R versus N

Lab-moderated models yielded nonsignificant effects of lab in all sets of data, as did the age-moderated models for age in sets 1 and 2. For set 3, age showed a trend toward significance, with the intercept being 0.19 for newborns ([−0.03, 0.40], z=1.72, p<0.1), and the decreases in effect magnitude were 0.07 for 6-month-olds, 0.36 for 7-month-olds, and 0.29 for 9-month-olds (Table S6 in the Supplementary Material). The model including repetition position as a moderator for set 3 yielded no significant effect.

#### 3.1.3 Estimates of bias

##### Funnel plots

**Fig. 5:**
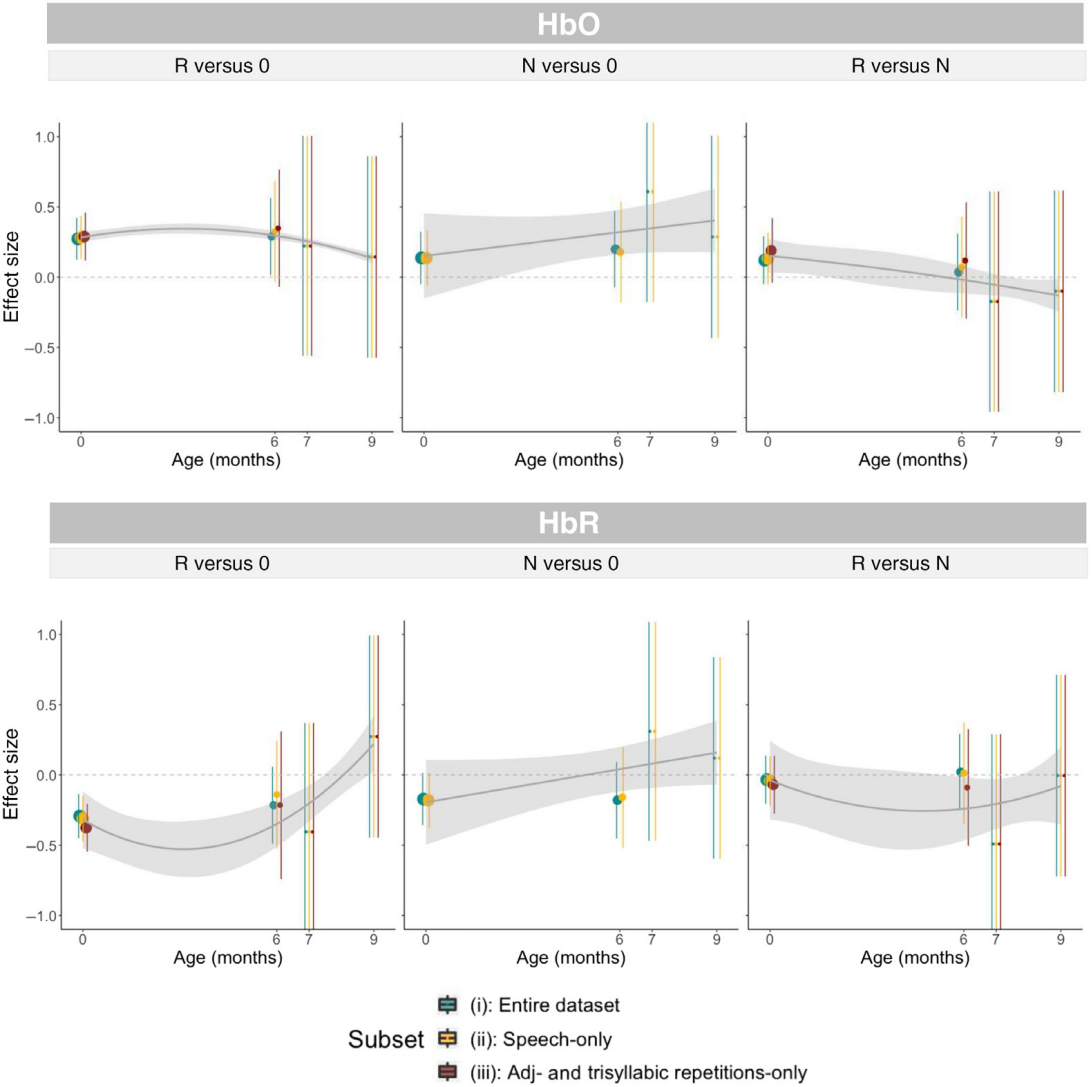
Funnel plots of effect sizes against standard errors, for each of the three comparisons of interest, obtained on HbO (top panel) and HbR time traces (bottom panel); the white region shows the 95% confidence interval around the estimates, and the gray region shows the 95% to 99% interval; colors indicate the lab where the study was carried out, and shapes indicate the age group of each study.

Figure 5 shows funnel plots for the three comparisons of interest over the entire dataset. In addition to visual inspection, we also carried out a rank correlation test^39^ to detect asymmetries in the funnel plots. Asymmetries were not detected in any comparison (HbO, R versus 0: Kendall’s τ=0.05, ns; N versus 0: Kendall’s τ=0.22, ns; R versus N: Kendall’s τ=0.04, ns; HbR, R versus 0: Kendall’s τ=0.11, ns; N versus 0: Kendall’s τ = 0.03, ns; R versus N: Kendall’s τ=0.11, ns).

##### Fail-safe-N

The estimated fail-safe-N for the R versus 0 comparison over set 1 was 126, indicating that 126 studies with a null effect would have to exist in the “file drawer” for the overall effect size to become zero. The fact that this number is much greater than the number of studies in the analysis suggests that the effect of repetition-based regularities on brain responses is robust.

### 3.2 Mixed-Effects Modeling

**Fig. 6:**
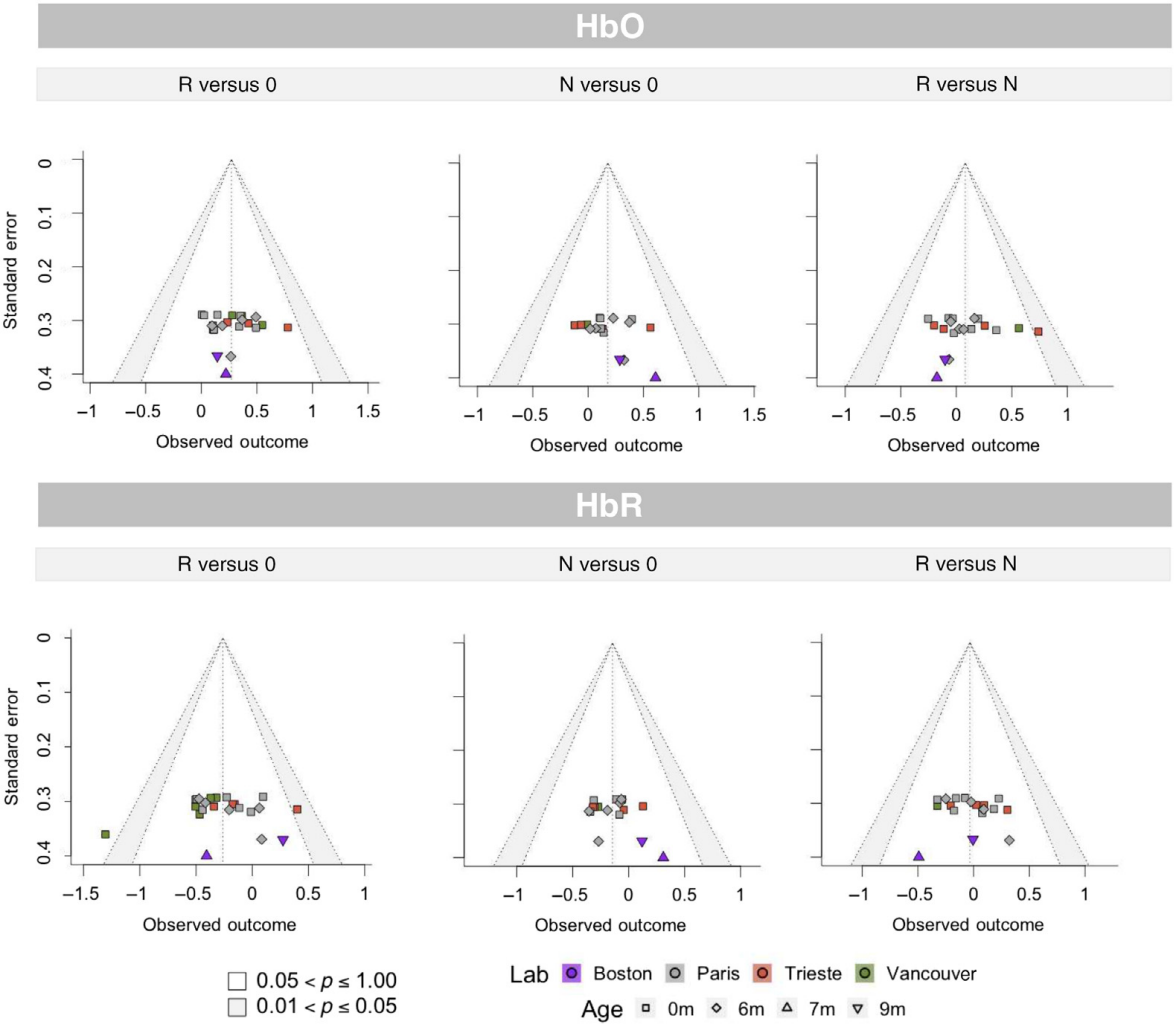
Distributions of individual effect sizes for the three comparisons of interest (R versus 0, N versus 0, and R versus N) for HbO (upper panel) and HbR (lower panel).

Distributions of individual effect sizes are shown in Fig. 6. Grand averages are shown in Fig. 8.

#### 3.2.1 Oxygenated hemoglobin (HbO)

For the R versus 0 comparison over set 1, the best fitting model included a random intercept for StudyID and fixed effects for lab and age, and it yielded a significant main effect of age (F(1, 410) = 3.96, p<0.05), carried by a marginally larger effect for 6-month-olds than for newborns (estimate: −0.166, t(19)=−1.97, p=0.06). Over set 2, the best-fitting model included a random intercept for StudyID and fixed effects for lab and age. This model also yielded a main effect of age (F(1,343)=5.48, p<0.05), carried by a larger effect in 6-month-olds than in newborns (estimate −0.27, t(26)=−2.33, p=0.02). Over set 3, the best-fitting model included a random intercept for StudyID and fixed effects for lab and age. The model yielded a significant main effect of age [F(1,266)=7.02, p<0.01], carried by a larger effect in 6-month-olds than in newborns [estimate −0.36, −(20)=−2.63, p=0.01].

For the N versus 0 comparison, for both sets 1 and 2, the best fitting models included a random intercept for StudyID and fixed effects for lab and age, but in both cases, no significant effects were found.

For the R versus N comparison, the best fitting models for sets 1 to 3 included a random intercept for StudyID and fixed effects for lab and age, but no significant effects were found.

#### 3.2.2 Deoxygenated hemoglobin (HbR)

For the R versus 0 comparison over set 1, the best fitting model included a random intercept for StudyID and fixed effects for lab and age, and it yielded a significant main effect of age (F(1,414)=6.34, p<0.05), carried by a stronger activation in 6-month-olds than in newborns (estimate 0.49, t(19)=2.49, p<0.05). A similar result was found for Set 2 (F(1,348=8.45,p<0.01), carried again by a larger effect size in 6-month-olds than in newborns (estimate 0.78, t(23)=2.89, p<0.01), as well as for set 3 (F(1,270)=11.95, p<0.001, estimate 0 to 6 month = 1.2, t(17)=3.43, p<0.01).

The best-fitting model comparing N versus 0 included a random intercept for StudyID and fixed effects for lab and age, but it yielded no significant effects for either set 1 or set 2.

For the R versus N comparison, the best fitting model similarly included a random intercept for StudyID and fixed effects for lab and age, but it did not yield significant effects for any of the sets of data.

## 4 Discussion

We have conducted meta-analyses and mixed-effects modeling-based inferential statistics to determine whether effect sizes were replicable in a sample of 23 NIRS studies investigating 0- to 9-month-old infants’ abilities to process repetition- and diversity-based regularities in linguistic and nonlinguistic auditory and visual sequences. In addition to quantifying effect sizes and their variation across studies, we have investigated whether these are modulated by different factors such as the age of participants or the laboratory, with the latter standing as a proxy for a set of various dimensions along which laboratories differed, e.g., NIRS machine, population characteristics, etc. We obtained NIRS data from 12 published and 11 unpublished studies. The 23 studies involved a total of 487 infants, aged between 0 and 9 months, tested in four different countries (Canada, France, Italy, and USA). The purpose of the study was primarily methodological, i.e., to test the replicability of experimental effects in infant NIRS research, an issue that has not been addressed in the developmental neuroscience literature, but which is essential for establishing NIRS as a reliable tool for infant brain imaging.

We tested three comparisons—infants’ responses to repetition-based regularities with respect to baseline (R versus 0), infants’ responses to diversity-based regularities with respect to baseline (N versus 0), and infants’ responses to repetition- versus diversity-based regularities (R versus N)—as all three are relevant to various aspects of language development.^26^

We used two statistical approaches: a meta-analytic one, which addresses variability and replicability at the study level, and linear mixed-effects modeling, which tests the significance of the factors of interest (study, lab, and age) over individual effect sizes, while taking into account the nested nature of sampling (study nested in lab) by the random effects structure. Importantly, in both cases, we used effect sizes (study-level effect sizes for meta-analyses and individual-level effect sizes for the mixed-effects models) and not hemoglobin concentrations as dependent variables. In other words, our analyses address not only whether infants respond to a certain structural regularity but also, and more importantly, whether and how the responses vary across studies and laboratories. If these factors are found to modulate effect sizes, then there is considerable methodological variation across studies, which may undermine replicability and raise concerns about the reliability of infant NIRS methodology.

### 4.1 Factors of Variability

#### 4.1.1 Cross-study variability and replicability

Our first and most important result is, therefore, that the factors/moderators study and laboratory never showed any significant effects for any of the comparisons in any of the analyses, meta-analytic or inferential. Effect sizes were statistically indistinguishable across different studies and laboratories. Importantly, this was true despite the fact that, as reported in the publications, studies and labs differed considerably in several factors known to impact NIRS data as well as along many undocumented factors that have less well known impacts on NIRS data (e.g., experimenter characteristics, features of the testing space, season/time of day of testing, etc.).

Estimates of bias, of which we computed the fail-safe-N and funnel plots, also confirm that the effects are robust and show no particular biases related to the selective publication of positive results or otherwise.

Our results, therefore, suggest that infants’ ability to process structural regularities in linguistic and nonlinguistic sequences can be tested in comparable and replicable ways across studies conducted with different NIRS machines, near-infrared light wavelengths, infants with different hair quality and color, etc. These results show, for the first time, that NIRS studies replicate robustly, even with the youngest infants.

#### 4.1.2 Theoretically relevant sources of variability

In addition to the above-discussed methodological factors, for which we expected no significant variation if infant NIRS studies were to be reliably replicable, we also included two additional factors in our analyses, age and repetition position, which have been suggested to modulate infants’ responses to structural regularities in theoretically relevant ways.

**Fig. 7:**
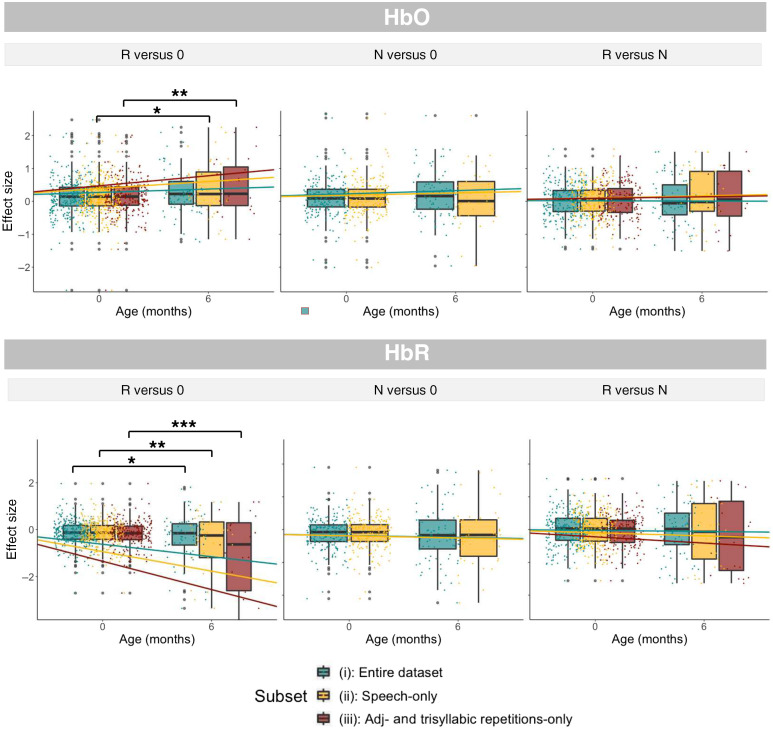
Meta-analytic effect sizes by age in sets 1 to 3. Gray bands indicate 95% confidence intervals, computed from the standard errors of each random-effects model. The top panel reports effect sizes obtained from HbO time traces, and the bottom panel shows the corresponding plots for HbR.

**Fig. 8:**
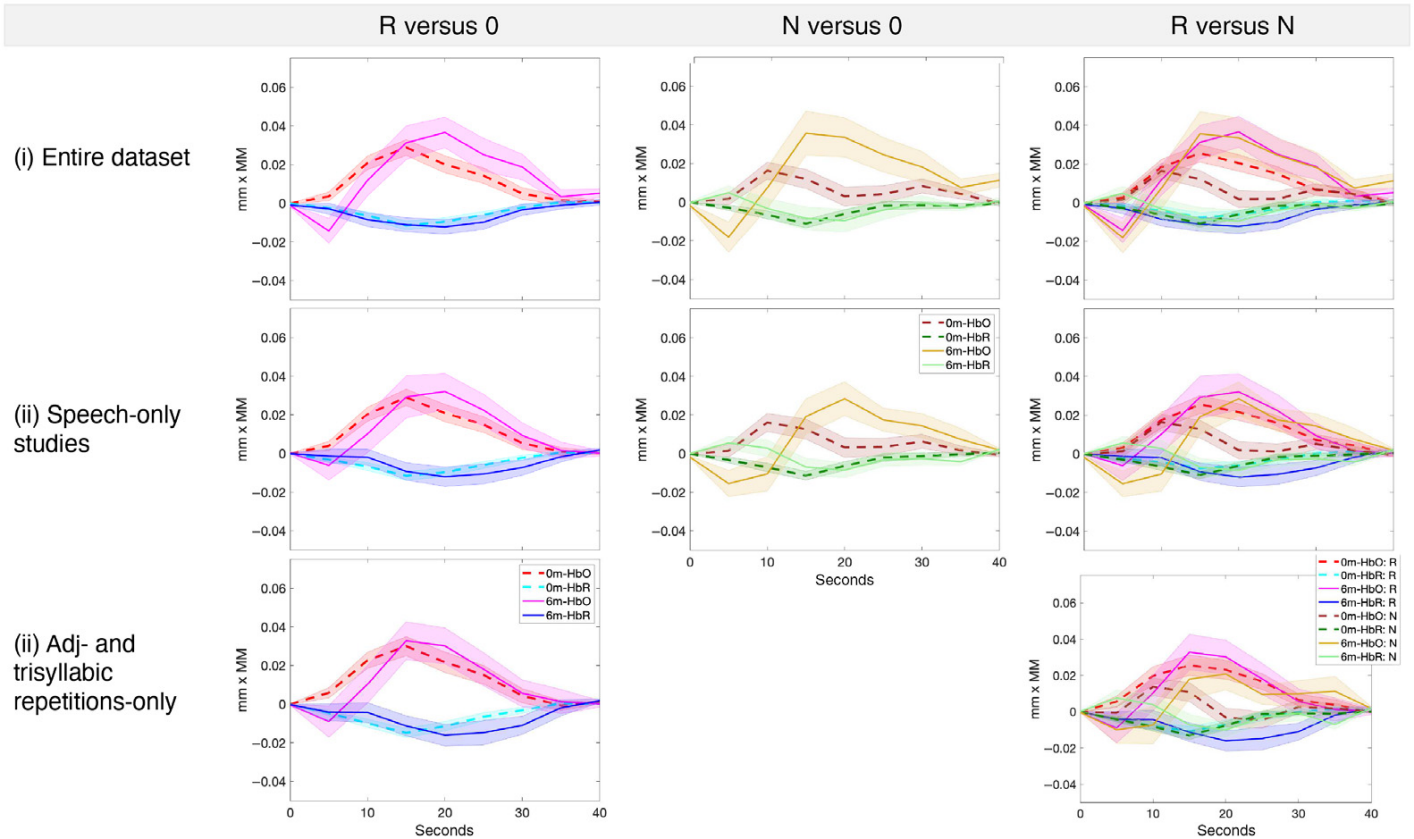
Grand average hemodynamic responses across studies in the left temporal area for the three comparisons of interest (R versus 0, N versus 0, and R versus N).

We found that age indeed modulated effect sizes. In the meta-analyses for the R versus N comparison, age showed a trend toward significance, with infants exhibiting a decreasing difference in their responses to repetition- versus diversity-based regularities with increasing age. These results are in accordance with the literature^26^ and derive from the response to repetition-based regularities remaining stable across developmental time with the response to diversity-based regularities increasing (Figs. 7 and 8). Although this tendency was numerically present in all sets of the data, it only approached significance in Set 3, i.e., studies using trisyllabic speech sequences with adjacent repetitions because, in this restricted set, variability attributable to other factors was reduced; thus the effect of age could surface more clearly. In the mixed-effects models, we also observed a significant main effect of age. However, in this case, we saw an age-related increase in the responses to repetition-based sequences compared with the baseline, with 6-month-olds’ effect sizes being greater than those of newborns. The effect was marginal over the entire set of studies, but became significant for sets 2 and 3, which were more homogeneous and thus had less variance. Although it may seem that the results of the meta-analyses and the mixed-effects models diverge, it needs to be remembered that the mixed-effects models did not include data from 7- and 9-month-olds, so this is only apparent given the lack of trial-by-trial data for those studies. As a result, the meta-analyses have detected a larger developmental trend, which the mixed-effects models could not find in the absence of relevant data. Relevantly, however, the increase in effect sizes between 0 and 6 months in the R versus 0 comparison is also present numerically in the meta-analyses, and indeed its magnitude increases as the sets of studies get more homogeneous.

We thus observed two developmental trends: a decrease in the differential response to repetition- versus diversity-based regularities, especially starting at 7 months, and an increase in the response to repetitions. Both were particularly strong for the least variable, most homogenous set of studies, which were those that tested adjacent repetitions in trisyllabic speech sequences. Whether similar trends may also be observed for other stimuli, e.g., for visual sequences, could not be determined as the number of studies using other types of stimuli was insufficient. The two observed developmental trends converge with existing findings as infants’ ability to detect repetitions has been shown to improve during the second half of the first year of life at the behavioral level,^35^^,^^36^ and the differential response between repetition-based and diversity-based patterns has been suggested to decrease by 6 months of age.^26^

By contrast, our results show no evidence for repetition position to modulate the effect sizes of infants’ responses in any of the analyses. This effect is indeed relatively weak, and has not systematically been found in all behavioral studies.^25^^,^^26^^,^^37^ We may thus not have sufficient statistical power to detect it as a lot fewer studies included in our sample tested initial repetition compared with the final ones, or the effect may genuinely be absent at the neural level.

### 4.2 Observed Effect Sizes

Our results have established that, at least for the linguistic tasks under investigation, effects obtained in infants using NIRS replicate across studies and labs, while they are modulated by developmental factors. It is thus now relevant to quantify the magnitude of these effects. Effect sizes are indeed becoming increasingly important statistical measures as experimental neuroscience moves away from the much criticized and highly restrictive interpretation of study results in terms of the strict dichotomy of significant and nonsignificant p-values.^40^^–^^42^

The effect size of infants’ responses to repetition-based regularities compared with the baseline was found to be close to 0.3, with only slight differences as a function of study sets and participants’ age, as discussed above. This is thus a moderate effect. Responses to diversity-based regularities tend to be weaker, varying between 0.14 and 0.20, i.e., they are small-to-moderate effects. The effects of the differential response between the two regularities show considerable variation and tend to be small, between 0.04 and 0.19.

Small-to-moderate effect sizes are quite common in the developmental literature, even in technically less challenging behavioral tasks.^23^^,^^33^ That infant neuroimaging, often presenting greater challenges both for researchers and participants, produces similar effect sizes can, therefore, be considered an important finding, demonstrating the reliability of infant NIRS methodology.

### 4.3 Limitations and Future Directions

Our study sought to quantify the sizes and variability of the effects found in a sample of NIRS studies with young infants to assess their replicability. Consequently, we needed to make some methodological choices to reduce the dimensionality of the data and to facilitate comparisons across studies. The most important of these was the decision to focus on an anatomically defined, predetermined region of interest (ROI), the left temporal area. This choice was motivated by various factors. First, this area has been shown to be strongly involved in speech and language processing from the earliest ages, as shown by converging evidence from various brain imaging modalities such as fMRI^43^ and NIRS.^44^ Second, this area showed the strongest activation in the first NIRS study testing the processing of repetition- and diversity-based regularities in newborns.^28^ Third, given the headgears and optode configurations used in the current studies, channels overlaid on the temporal areas seemed to vary less with respect to the underlying brain anatomy than other channels, even despite age-related changes in head and brain size, as shown by localization analyses.^31^^,^^32^ This region of interest is thus useful for obtaining comparable data across many different studies and so was well suited for our purposes. However, for a more theoretically oriented meta-analysis, i.e., if the goal is to better understand the neural mechanisms underlying infants’ rule learning abilities, it will be more suitable to derive functionally-based, data-driven ROIs specifically for each study as it may be the case that the strongest effects are found in different ROIs in different studies, e.g., as a function of age-related changes or changes in the nature or sensory modality of the stimuli. Using such an approach is beyond the scope of the current study, but future research may address this issue.

## 5 Conclusion

The replication crisis has raised serious questions about the reliability and robustness of empirical findings in many disciplines from sociology and economics to psychology and neuroscience. NIRS, being a relatively recent brain imaging technique, with little standardization in research and analysis practices across laboratories, is in need of a systematic assessment of study replicability. In a meta-analysis of 23 NIRS studies on infants’ rule-learning abilities, we have shown, for the first time, that effects are robust and replicable across different studies and laboratories, with small-to-moderate effect sizes, as is typical in developmental research. This approach also allowed us to identify moderating factors relevant to theory-building, such as developmental age.
