# Reproducibility of infant fNIRS studies: a meta-analytic approach (Erratum)

**DOI:** 10.1117/1.NPh.10.2.029801

**Published:** 2023-04-04

**Authors:** Jessica Gemignani, Irene de la Cruz-Pavía, Anna Martinez, Caroline Nallet, Alessia Pasquini, Gaia Lucarini, Francesca Cavicchiolo, Judit Gervain

**Affiliations:** a University of Padua, Department of Developmental and Social Psychology, Padua, Italy; b University of Padua, Padova Neuroscience Center, Padua, Italy; c University of the Basque Country, Department of Linguistics and Basque Studies, Vitoria-Gasteiz, Spain; d Ikerbasque, Basque Foundation for Science, Bilbao, Spain; e Université Paris Cité & CNRS, Integrative Neuroscience and Cognition Center, Paris, France

## Abstract

The erratum documents correction of erroneous captions for Figs. 5-7.

This article [*Neurophotonics* **10**(2), 023518 (2023) doi: https://doi.org/10.1117/1.NPh.10.2.023518] was originally published on 8 March 2023 with erroneous captions for Figs. 5–7.

• Figure 5 was published with the caption for Fig. 6
• Figure 6 was published with the caption for Fig. 7
• Figure 7 was published with the caption for Fig. 5

The captions were correctly re-assigned, and the article was republished on 28 March 2023.

