# Supplementary material for: Reproducibility of infant fNIRS studies: a meta-analytic approach
Source: Neurophotonics. 2023 Mar 8;10(2):023518. doi: 10.1117/1.NPh.10.2.023518 (PMC9997722; doi:10.1117/1.NPh.10.2.023518)
Supplement: Supplementary file 1 [file NPh_010_023518_SD001.pdf]

The reproducibility of infant fNIRS studies: a meta-analytic approach

Jessica Gemignani [a,b*], Irene de la Cruz-Pavía [c,d], Anna Martinez [a,b], Caroline Nallet [a,b], Alessia Pasquini [a], Gaia Lucarini [a,b], Francesca Cavicchiolo [a], Judit Gervain[a,b,e]

[a] University of Padua, Department of Developmental and Social Psychology, Padua, Italy
[b] University of Padua, Padova Neuroscience Center, Padua, Italy
[c] University of the Basque Country, Department of Linguistics and Basque Studies, Vitoria-Gasteiz, Spain
[d] Ikerbasque, Basque Foundation for Science, Bilbao, Spain
[e] Université Paris Cité & CNRS, Integrative Neuroscience and Cognition Center, Paris, France

Supplementary Material

3. RESULTS

3.1 Meta-analysis

3.1.1 Unmoderated models

Figure S1 shows the forest plots obtained on the HbR timetraces for the three comparisons of interest (R vs 0, N vs 0, R vs N).

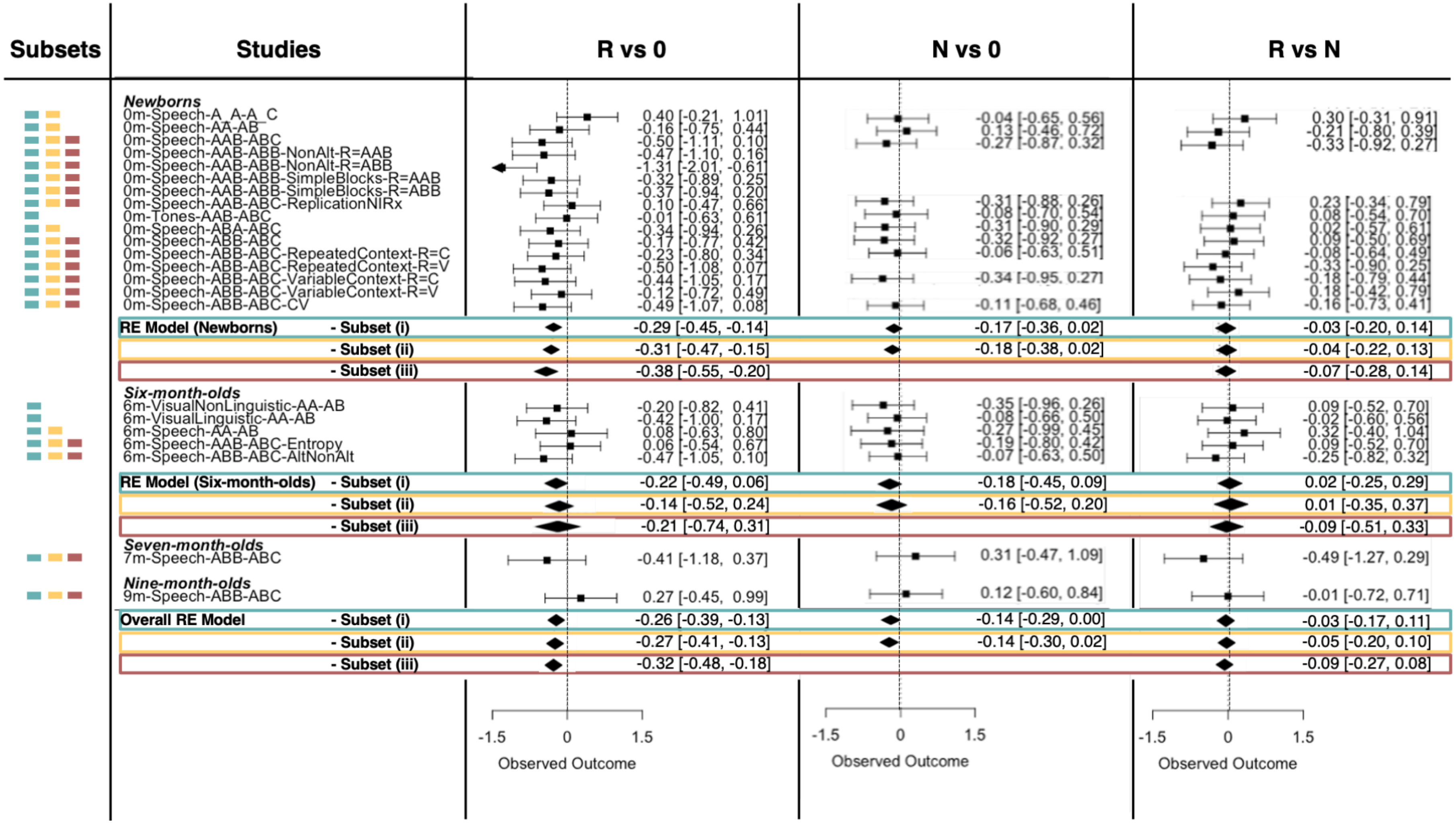

Figure S1: Forest plots of the meta-analytic effect sizes for brain activation in the left temporal area to repetition-based- (R vs 0) and diversity-based regularities (N vs 0), as well as for the difference in activation between them (R vs N). Each study's estimate is indicated by the corresponding square. Error bars indicate the 95% Confidence Interval. Diamonds show the summary estimates of each subset of studies, with the center of the diamond corresponding to the estimate and the left and right edges indicating the confidence interval limits. Not all studies contributed to all comparisons, as described in Section 2.1.1 and Table 1.

3.1.2 Moderated analyses

The following tables S1-S8 report model outputs for each moderated analysis (Age-, Lab- and Repetition Position-moderated models), carried out on HbO and HbR independently.

R vs 0

- **Age-moderated models (Table S1)**

	Set 1							
	HbO				HbR			
	Est	SE	*Z*	*p*	Est	SE	*Z*	*p*
**Intercept (0m)**	0.2731	0.0757	3.6081	**0.0003**	-0.2923	0.0761	-3.8389	**0.0001**
**Age6m**	0.0162	0.1590	0.1018	0.9189	0.0766	0.1594	0.4807	0.6307
**Age7m**	-0.0513	0.4066	-0.1262	0.8996	-0.1139	0.4037	-0.2822	0.7778
**Age9m**	-0.1288	0.3734	-0.3448	0.7303	0.5655	0.3747	1.5092	0.1313
	**Set 2**							
	**HbO**				**HbR**			
	**Est**	**SE**	***Z***	***p***	**Est**	**SE**	***Z***	***p***
**Intercept (0m)**	0.2731	0.0757	3.6081	**0.0003**	-0.2923	0.0761	-3.8389	0.0001
**Age6m**	0.0162	0.159	0.1018	0.9189	0.0766	0.1594	0.4807	0.6307
**Age7m**	-0.0513	0.4066	-0.1262	0.8996	-0.1139	0.4037	-0.2822	0.7778
**Age9m**	-0.1288	0.3734	-0.3448	0.7303	0.5655	0.3747	1.5092	0.1313
	**Set 3**							
	**HbO**				**HbR**			
	**Est**	**SE**	***Z***	***p***	**Est**	**SE**	***Z***	***p***
**Intercept (0m)**	0.2892	0.0869	3.3293	**0.0009**	-0.3757	0.0875	-4.2944	<.0001
**Age6m**	0.0589	0.2299	0.2564	0.7977	0.1554	0.2299	0.6758	0.4991
**Age7m**	-0.0675	0.4088	-0.165	0.8689	-0.0305	0.406	-0.0751	0.9402
**Age9m**	-0.1449	0.3759	-0.3856	0.6998	0.649	0.3772	1.7205	0.0853

- **Lab-moderated models (Table S2)**

	Set 1							
	HbO				HbR			
	Est	SE	*Z*	*p*	Est	SE	*Z*	*p*
**Intercept (Boston)**	0.1796	0.2697	0.6659	0.5055	-0.0402	0.2693	-0.1494	0.8812
**Paris**	0.0581	0.2837	0.2046	0.8379	-0.1927	0.2833	-0.6802	0.4964
**Trieste**	0.2024	0.3105	0.652	0.5144	-0.0328	0.3096	-0.1059	0.9156
**Vancouver**	0.1081	0.3021	0.3578	0.7205	-0.5052	0.303	-1.6674	0.0954

	Set 2							
	HbO				HbR			
	Est	SE	*Z*	*p*	Est	SE	*Z*	*p*
**Intercept (Boston)**	0.1796	0.2697	0.6659	0.5055	-0.0402	0.2693	-0.1494	0.8812
**Paris**	0.0708	0.2881	0.2458	0.8058	-0.1973	0.2877	-0.6859	0.4928
**Trieste**	0.2024	0.3105	0.652	0.5144	-0.0328	0.3096	-0.1059	0.9156
**Vancouver**	0.1081	0.3021	0.3578	0.7205	-0.5052	0.303	-1.6674	0.0954
	**Set 3**							
	**HbO**				**HbR**			
	**Est**	**SE**	***Z***	***p***	**Est**	**SE**	***Z***	***p***
**Intercept (Boston)**	0.1796	0.2697	0.6659	0.5055	-0.0402	0.2693	-0.1494	0.8812
**Paris**	0.0695	0.2896	0.2401	0.8102	-0.224	0.2892	-0.7745	0.4386
**Trieste**	0.5987	0.413	1.4497	0.1471	-0.1342	0.4048	-0.3316	0.7402
**Vancouver**	0.1081	0.3021	0.3578	0.7205	-0.5052	0.303	-1.6674	0.0954

- **Repetition Position – moderated model (Table S3)**

	Set 3							
	HbO				HbR			
	Est	SE	*Z*	*p*	Est	SE	*Z*	*p*
**Intercept (Initial)**	0.2527	0.1351	1.8706	0.0614	-0.2174	0.1353	-1.6074	0.108
**Final**	0.0523	0.1645	0.3179	0.7505	-0.1636	0.165	-0.9912	0.3216

N vs 0

- **Age-moderated models (Table S4)**

	Set 1							
	HbO				HbR			
	Est	SE	*Z*	*p*	Est	SE	*Z*	*p*
**Intercept (0m)**	0.1367	0.0954	1.4321	0.1521	-0.1716	0.0953	-1.799	0.0719
**Age6m**	0.062	0.1689	0.3671	0.7135	-0.0088	0.1687	-0.052	0.9583
**Age7m**	0.4724	0.4125	1.1452	0.2521	0.4817	0.4084	1.1794	0.2382
**Age9m**	0.1499	0.3793	0.3953	0.6926	0.2916	0.3786	0.7703	0.4412
	**Set 2**							
	**HbO**				**HbR**			
	**Est**	**SE**	***Z***	***p***	**Est**	**SE**	***Z***	***p***

**Intercept (0m)**	0.1364	0.1001	1.3625	0.1731	-0.1805	0.1	-1.805	0.071
**Age6m**	0.042	0.209	0.2009	0.8408	0.0211	0.2086	0.1013	0.9193
**Age7m**	0.4727	0.4136	1.1429	0.2531	0.4905	0.4095	1.1979	0.231
**Age9m**	0.1502	0.3805	0.3949	0.6929	0.3004	0.3797	0.7912	0.4288

- **Lab-moderated models (Table S5)**

	**Set 1**							
	**HbO**				**HbR**			
	**Est**	**SE**	***Z***	***p***	**Est**	**SE**	***Z***	***p***
**Intercept (Boston)**	0.4335	0.2708	1.6005	0.1095	0.2074	0.2693	0.7702	0.4412
**Paris**	-0.247	0.2875	-0.859	0.3904	-0.3874	0.286	-1.3546	0.1755
**Trieste**	-0.31	0.311	-0.9967	0.3189	-0.343	0.3093	-1.1089	0.2675
**Vancouver**	-0.4424	0.4054	-1.0914	0.2751	-0.48	0.4054	-1.1838	0.2365
	**Set 2**							
	**HbO**				**HbR**			
	**Est**	**SE**	***Z***	***p***	**Est**	**SE**	***Z***	***p***
**Intercept (Boston)**	0.4335	0.2708	1.6005	0.1095	0.2074	0.2693	0.7702	0.4412
**Paris**	-0.2523	0.2942	-0.8576	0.3911	-0.3918	0.2927	-1.3385	0.1807
**Trieste**	-0.31	0.311	-0.9967	0.3189	-0.343	0.3093	-1.1089	0.2675
**Vancouver**	-0.4424	0.4054	-1.0914	0.2751	-0.48	0.4054	-1.1838	0.2365

R vs N

- **Age-moderated models (Table S6)**

	**Set 1**							
	**HbO**				**HbR**			
	**Est**	**SE**	***Z***	***p***	**Estimate**	**SE**	***Z***	***p***
**Intercept (0m)**	0.1209	0.0872	1.387	0.1654	-0.0335	0.0869	-0.386	0.6998
**Age6m**	-0.0849	0.164	-0.5176	0.6047	0.0535	0.1642	0.3258	0.7445
**Age7m**	-0.2954	0.4093	-0.7217	0.4705	-0.4582	0.408	-1.123	0.2614
**Age9m**	-0.2213	0.3761	-0.5884	0.5562	0.0281	0.3758	0.0749	0.9403
	**Set 2**							
	**HbO**				**HbR**			
	**Est**	**SE**	***Z***	***p***	**Estimate**	**SE**	***Z***	***p***
**Intercept (0m)**	0.1326	0.0907	1.4625	0.1436	-0.0426	0.0904	-0.471	0.6376
**Age6m**	-0.0607	0.2043	-0.2972	0.7663	0.0548	0.2046	0.2679	0.7888

**Age7m**	-0.3071	0.4101	-0.7489	0.4539	-0.4492	0.4087	-1.099	0.2718
**Age9m**	-0.233	0.3769	-0.6182	0.5364	0.0372	0.3767	0.0988	0.9213
	**Set 3**							
	**HbO**				**HbR**			
	**Est**	**SE**	***Z***	***p***	**Estimate**	**SE**	***Z***	***p***
**Intercept (0m)**	0.1884	0.1094	1.7212	0.0852	-0.0705	0.1053	-0.669	0.5036
**Age6m**	-0.0704	0.2445	-0.2882	0.7732	-0.0198	0.2366	-0.083	0.9333
**Age7m**	-0.3628	0.422	-0.8598	0.3899	-0.4213	0.4123	-1.021	0.3068
**Age9m**	-0.2887	0.3898	-0.7407	0.4589	0.0651	0.3805	0.171	0.8642

- **Lab-moderated models (Table S7)**

	**Set 1**							
	**HbO**				**HbR**			
	**Est**	**SE**	***Z***	***p***	**Est**	**SE**	***Z***	***p***
**Intercept (Boston)**	-0.1341	0.2699	-0.4969	0.6193	-0.2276	0.2694	-0.8448	0.3982
**Paris**	0.1717	0.2838	0.605	0.5452	0.2119	0.2834	0.7478	0.4546
**Trieste**	0.2970	0.3105	0.9566	0.3388	0.2777	0.3094	0.8974	0.3695
**Vancouver**	0.6974	0.4091	1.7046	0.0883	-0.0975	0.4059	-0.2402	0.8102
	**Set 2**							
	**HbO**				**HbR**			
	**Est**	**SE**	***Z***	***p***	**Est**	**SE**	***Z***	***p***
**Intercept (Boston)**	-0.1341	0.2699	-0.4969	0.6193	-0.2276	0.2694	-0.8448	0.3982
**Paris**	0.1890	0.2881	0.6558	0.5120	0.1921	0.2878	0.6676	0.5044
**Trieste**	0.2970	0.3105	0.9566	0.3388	0.2777	0.3094	0.8974	0.3695
**Vancouver**	0.6974	0.4091	1.7046	0.0883	-0.0975	0.4059	-0.2402	0.8102
	**Set 3**							
	**HbO**				**HbR**			
	**Est**	**SE**	***Z***	***p***	**Est**	**SE**	***Z***	***p***
**Intercept (Boston)**	-0.1341	0.2699	-0.4969	0.6193	-0.2276	0.2694	-0.8448	0.3982
**Paris**	0.1989	0.2896	0.687	0.4921	0.1630	0.2892	0.5636	0.5730
**Trieste**	0.8733	0.4139	2.1096	0.0349	0.3209	0.4047	0.7929	0.4278
**Vancouver**	0.6974	0.4091	1.7046	0.0883	-0.0975	0.4059	-0.2402	0.8102

- **Repetition Position – moderated model (Table S8)**

	**Set 3**	
	**HbO**	**HbR**

	Est	SE	*Z*	*p*	Est	SE	*Z*	*p*
**Intercept (Initial)**	0.1762	0.1741	1.012	0.3116	0.0032	0.1736	0.0184	0.9853
**Final**	-0.0495	0.2028	-0.244	0.8072	-0.128	0.2022	-0.6327	0.5269
